# Diabetes‐Related Foot Disease in South Asians Living in Western Countries: Burden, Outcomes, and Gaps in the Literature—A Scoping Review Protocol

**DOI:** 10.1111/iwj.70772

**Published:** 2025-11-02

**Authors:** Uroosa R. Khan, David A. Russell

**Affiliations:** ^1^ Department of Podiatry Leeds Teaching Hospitals NHS Trust Leeds UK; ^2^ Leeds NIHR Biomedical Research Centre Leeds Teaching Hospitals NHS Trust Leeds UK; ^3^ Leeds Institute for Clinical Trials Research, University of Leeds Leeds UK

**Keywords:** amputation, diabetes mellitus, diabetic feet, scoping review, wounds

## Abstract

In England, diabetes‐related foot ulcers and related amputations equate to approximately 1% of the National Health Service budget. Most of these costs are related to hospital admissions with diabetes‐related foot ulcers, found to be 8.04 days longer when compared to those without ulcers. Although South Asian (SA) populations living in Western countries experience disproportionately high diabetes rates, they exhibit significantly lower prevalence of diabetes‐related foot ulcers and lower‐limb amputations compared to White European populations. This paradox remains underexplored, necessitating a scoping review to map existing evidence, elucidate disparities, and identify gaps. To explore the burden of diabetes‐related foot ulcers among South Asians living in Western countries by examining their incidence, prevalence, and predisposing factors. Assessing clinical outcomes and lived experiences during the ulcerative phase and reviewing existing literature on recurrence and long‐term post‐healing complications. Following Joanna Briggs Institute methodology and reported in line preferred reporting items for systematic reviews and meta‐analyses extension for scoping reviews. A comprehensive search will be conducted across databases, and registered with open science framework. This will be the first scoping review to map diabetes‐related foot ulcers burden among South Asians in Western settings. Clarifying incidence and outcome disparities, highlighting research gaps, and suggesting directions for future studies.

**Summary**

- What is already known?
  ○ South Asians living in Western countries have higher diabetes prevalence, but paradoxically lower rates of diabetic foot ulcers (DFUs) and amputations compared to other ethnic groups.
- What this study has found?
  ○ This will be the first scoping review to comprehensively map and synthesise evidence on the incidence, burden, and outcomes of DFUs in South Asians in Western contexts.
- What are the implications?
  ○ This will be the first scoping review to comprehensively examine the burden of diabetic foot ulcers among South Asians in Western settings. It will clarify disparities in incidence and outcomes, highlight key research gaps, and provide valuable insights to inform future research and clinical practice.

## 1 Introduction

Diabetes‐related foot ulcers (DFUs) are among the most severe and costly complications of diabetes, posing challenges to healthcare systems and patients worldwide. The pathway to ulceration is typically driven by a combination of peripheral arterial disease, neuropathy, and poor glycaemic control [1]. These factors collectively impair wound healing, predisposing patients to chronic or recurrent ulceration. This is associated with serious clinical outcomes, including infection, hospitalisation, and the need for both minor and major lower limb amputations [2]. Current evidence indicates that individuals with diabetes are five times more likely to undergo lower‐limb amputation compared to those without diabetes, leading to substantial morbidity, reduced quality of life, and increased mortality [3, 4].

With this in mind, DFUs pose a significant economic burden on health care systems worldwide. In the United Kingdom, treatment of DFUs equates to approximately 1% of the National Health Service (NHS) England's budget. Most of these costs were related to hospital admissions, with DFU cases found to be 8.04 days longer when compared to those without ulcers [5].

While the financial costs are considerable, it could be argued that the burden of DFUs is not distributed equally across all populations. It has been found that South Asians (SA), particularly those of Pakistani, Bangladeshi, and Indian descent, have a disproportionately high prevalence of diabetes among European ethnic groups [6]. Studies in Canada showed that the highest incidence of type 2 diabetes was found in SA when compared to Chinese, White, and Black populations [7]. This phenomenon was initially found in the SA diaspora communities that had migrated to higher‐income countries [8]. Interestingly, despite the elevated diabetes burden, the SA population exhibits the lowest rates of lower‐limb amputation when compared to their White and Black counterparts [9]. A cohort study in New Zealand found that SA had a significantly reduced risk of lower limb amputation when compared to European cohorts [10]. Similar research in the United Kingdom reported that SA had one‐third the risk of developing a DFU compared to Europeans and was hypothesised to be due to lower rates of peripheral arterial disease and neuropathy in this group [11]. Furthermore, a 10‐year cohort study in Singapore by Riandini et al. following 156 593 participants found that South Asian Indians had significantly lower rates of diabetes‐related foot ulcers and lower extremity amputations compared to East Asians, despite having a higher prevalence of diabetes [12].

Currently, limited research exists to explain this disparity, which is likely shaped by more than genetic predispositions, involving a complex interplay of biological, social, cultural, and health system [13]. Lifestyle‐related risks such as central obesity, physical inactivity, and the adoption of westernised dietary patterns may increase diabetes risk [14, 15]. However, differences in the development of diabetes‐related complications may be shaped by factors, including PAD, neuropathy, footwear practices, health‐seeking behaviours, and access to foot care services [16, 17]. While genetic predispositions cannot be ruled out and may play a role, the lower observed rates of DFUs and amputations in South Asians are more likely explained by this multifactorial interaction.

By systematically mapping out existing literature, this review will help provide direction on potential explanations for these disparities and highlight gaps in knowledge. Identifying these factors could help inform targeted strategies such as culturally tailored education, early screening initiatives, and improved timely access to podiatry services as strategies to reduce the burden of diabetic foot complications in the South Asian population residing in western countries.

As a result, a scoping review has been proposed to explore this phenomenon in greater depth and contribute to a more comprehensive understanding of the prevalence of diabetic foot disease and its burdens to the SA population, and how outcomes differ when compared to other ethnicities.

### 1.1 Rationale

A prior protocol has been written to reduce bias, enable transparency, and increase the reproducibility of this scoping review. This protocol was written following the Joanne Briggs Institute (JBI) guidance and reporting items for the development of scoping review protocols [18]. A scoping review enables a comprehensive synthesis of the broader evidence base and was considered the most appropriate methodology for addressing the aims of this review. The burden of diabetes‐related foot disease within the SA population remains insufficiently explored. To our knowledge, there have been no scoping reviews on this topic. Our preliminary search did find an abstract reporting a systematic review examining prevalence, progression, and outcomes of DFUs and lower limb amputations in SA communities, although this has not since been published as a peer‐reviewed full manuscript [19]. Therefore, this scoping review will provide a broad search into this topic area and provide a comprehensive insight into factors such as the prevalence and outcomes of diabetes‐related foot disease on the SA population.

### 1.2 Deviations From the Protocol

This protocol is structured according to the steps outlined by preferred reporting items for systematic reviews and meta‐analyses extension for scoping reviews (PRISMA‐ScR) and the JBI framework. As this is a scoping review, deviation from the protocol will be expected. All deviations will be tracked on the review's open science framework project page and clearly outlined in the final report to ensure full transparency.

## 2 Aim

To explore the burden of diabetic foot ulcers (DFUs) among South Asians living in Western countries by examining their incidence, prevalence, and predisposing factors. We will assess clinical outcomes and lived experiences during the ulcerative phase and review existing literature on recurrence and long‐term post‐healing complications.

## 3 Method

The review will be conducted in accordance with JBI methodology for scoping reviews, and the reporting will follow the PRISMA‐ScR guidelines [20].

### 3.1 Key Definitions and Conceptional Clarifications

This review will use the following key definitions to ensure consistency in literature searches, data extraction, and interpretation.

*South Asian*: using the United Nations definition which includes Afghanistan Bangladesh, Bhutan, India, Iran, the Maldives, Nepal, Pakistan, and Sri Lanka [21].

*Western world/The west**:*** countries in western Europe or countries originating from Western European settlers. Specifically, this includes:

– *Western Europe*: countries such as the United Kingdom, France, Germany, Italy, Spain, Netherlands, Belgium, Switzerland, Austria, Denmark, Finland, Iceland, Norway, and Sweden
– *North America*: The United States and Canada.
– *Australasia*: Australia and New Zealand [22].

*Diabetes‐related foot ulcer (DFU)*: A foot ulcer penetrating below the dermis into subcutaneous structures, such as the fascia, muscle, tendon, or bone [23].

*Diabetes‐related foot disease*: Disease of the foot of a person with current or previously diagnosed diabetes mellitus that includes one or more of the following: peripheral neuropathy, peripheral artery disease, infection, ulcer(s), neuro‐osteoarthropathy, gangrene, or amputation [23].

### 3.2 Identifying the Research Question

We have used the JBI population, concept and context (PCC) framework approach to develop the research question for this scoping review. For the current review, the South Asian individuals living in the west (P) with diabetic foot ulcers (C) and exploring pre‐dispositions, ulcerative, and post healing phases (C) see Table 1.

**TABLE 1:** PCC framework.

Population	Concept	Context
South Asian individuals living in western counties with diabetes	Diabetic foot ulcers (DFU)	Predispositions: incidence and prevalence of DFU Ulcerative stage: burden of DFU, wound healing and time to healing Post‐healing phase: recurrence and long‐term complications, including minor and major amputations, health‐related quality of life, and mortality

Both the PCC framework, inclusion and exclusion criteria have been guided by the recently published core outcome set (COS) for studies evaluating interventions for diabetes‐related foot ulceration. Developed using the core outcome measures in effectiveness trials (COMET) methodology by Staniszewska et al., this COS was designed to improve consistency in outcome reporting across studies [24]. It identifies eight key outcomes: wound healing, time to healing, new or recurrent ulceration, infection, major amputation, minor amputation, health‐related quality of life, and mortality. These outcomes will inform the screening process, enhancing the comparability of extracted data and strengthening the overall validity of this review.

Research Question:

1. What is the incidence and prevalence of diabetes‐related foot ulcers among South Asians living in Western countries, and what factors predispose this population to ulceration?
2. What are the burdens and lived experienced by South Asian individuals during the ulcerative stage of diabetes related foot disease?
3. What is known about post‐healing outcomes in South Asian individuals, including recurrence rates and long‐term complications following diabetes‐related foot ulceration?

### 3.3 Identification of Relevant Studies

*Eligibility criteria*: The PCC framework will be used to help inform the inclusion and exclusion criteria, see Table 2 [13]. The review will only include research papers with full text available in English and consider both qualitative, quantitative, and mixed methods. To capture all papers available on this topic, no publication time range will be set. It will also include unpublished (grey) literature.

**TABLE 2:** Eligibility criteria.

PCC framework	Inclusion criteria	Exclusion criteria	Justification
Population	– Publications that include participants that are SA and have diabetes living in the west	– Any publication that does not include SA with diabetes	– The objective of this study is to investigate SA with diabetes
Concept	– Publication that reports on diabetic foot disease – Publication that reports on diabetic foot ulcers	– Any publication that explores diabetic foot disease or DFU but does not include SA	– The objective of this study is to investigate DFU/diabetic foot disease in SA
Context	– Publications that report on the predisposing factors of DFU – Publications that report on outcomes of DFU. Specifically minor and major amputations – Publications that report on experiences of having DFUs	– Any publication that does not look at DFU	– The objective of this study is to investigate incidence, prevalence, and predisposing factors of DFU in SA communities – The objective of this study is to look at the burdens of DFU on SA – The objective of this study is to understand the post healing or long‐standing complications of DFUs on SA people

### 3.4 Search Strategy

In keeping with the JBI recommendations, an initial limited search using the PCC framework ideas will be expanded to help develop search terms. This will be used over two databases: MEDLINE and EMBASE. Initial analysis will be of text words from titles, abstracts, and index terms retrieved from articles, followed by a comprehensive second search using all the identified keywords and terms across all databases. The reference lists of all identified sources will be screened to identify additional relevant literature.

The search process will be iterative, allowing refinement of terms and sources as familiarity with the literature grows, with all modifications or deviations documented transparently to Open Science Framework to ensure reproducibility. The development and peer review of the search strategy will involve the expertise of a research librarian. An example of search terms used in the initial search has been added; see Table 3.

**TABLE 3:** A table of initial Boolean search terms used.

Category	Line(s)	Search term
South Asian population	1–10	exp south asian/ south* asia*.tw,kf. indian*.tw,kf. bangladeshi*.tw,kf. afghan*.tw,kf. pakistani*.tw,kf. bhutan*.tw,kf. nepal*.tw,kf. maldiv*.tw,kf. sri lankan*.tw,kf. or/1‐10
Diabetes	12–15	12 diabet*.tw,kf. 13 T1D*.tw,kf. 14 T2D*.tw,kf. 15 exp. diabetes mellitus/ 16 or/12‐15
Foot conditions	17–24	17 foot.tw,kf. 18 feet.tw,kf. 19 plantar*.tw,kf. 20 podiat*.tw,kf. 21 exp foot ulcer/ 22 foot amputation/ 23 exp foot/ 24 podiatry/ 25 or/17‐24
Diabetes + foot	26–28	26 16 and 25 27 exp diabetic foot/ 28 26 or 27
Geography and population	29–48	29 exp United Kingdom/ 30 high income country/ 31 developed country/ 32 immigrant*.tw,kf. 33 undocumented immigrant/ 34 exp migrant/ 35 ancestry group/ 36 ethnic group/ 37 minority group/ 38 minority health/ 39 (emigrant* or immigrant* or migrant*).tw,kf. 40 (minorit* adj3 (population* or health or group*)).tw,kf. 41 exp western europe/ 42 exp North America/ 43 exp ‘Australia and New Zealand’/ 44 (UK or united kingdom or britain or british or england or scotland or scottish or wales or welsh or ireland or irish or NHS or national health service or london or manchester or glasgow or edinburgh or newcastle or leeds or birmingham or belfast or cardiff or bristol or sheffield or york*).tw,kf. 45 (europe* or france or french or belgium or belgian or netherlands or dutch or denmark or danish or german* or spain or spanish or portug* or italy or italian or austria* or sweden or swedish or norway or norwegian or finland or finnish or scandinavia* or nordic).tw,kf.
		(canada or Canadian or USA or united states or north america* or Alaska or Arizona or Arkansas or ‘American Samoa’ or California or Colorado or Connecticut or Delaware or ‘District of Columbia’ or Florida or Georgia or Guam or Hawaii or Idaho or Illinois or Indiana or Iowa or Kansas or Kentucky or Louisiana or Maine or Maryland or Massachusetts or Michigan or Minnesota or Mississippi or Missouri or Montana or Nebraska or Nevada or ‘New Hampshire’ or ‘New Jersey’ or ‘New Mexico’ or ‘New York’ or ‘North Carolina’ or ‘North Dakota’ or ‘Northern Mariana Islands’ or Ohio or Oklahoma or Oregon or Pennsylvania or ‘Puerto Rico’ or ‘Rhode Island’ or ‘South Carolina’ or ‘South Dakota’ or Tennessee or Texas or Utah or Vermont or Virginia or ‘Virgin Islands’ or Washington or ‘West Virginia’ or Wisconsin or Wyoming).tw,kf. (australia* or new zealand or sydney or canberra or melbourne or queensland or victoria* or brisbane or perth or cairns or hoabrt or adelaide or auckland or christchurch or wellington).tw,kf. ((western or developed or high income or OECD) adj5 (countr* or nation* or region*)).tw,kf. or/29–48
Final combined search	50	11 and 28 and 49

### 3.5 Study Selection

Study selection will be conducted in two stages: screening titles and abstracts, followed by full text reviews. Two independent, blinded reviewers will screen all sources for eligibility based on predefined inclusion criteria guided by the PCC framework, using Rayyan to manage the screening process. Any disagreements will be resolved through consensus or by involving a third reviewer.

Prior to the full screening, a pilot test on a random sample of 25 records to ensure consistency among reviewers will be performed [18]. Discrepancies will be discussed, and the eligibility criteria will be refined based on this. The screening will start once 75% agreement is achieved.

The selection process will be reported using the PRISMA‐ScR flowchart [20].

### 3.6 Data Collection

In line with the JBI and PRISMA–ScR guidelines, this review will employ a structured data reporting format. A standardised data charting form will be developed and piloted, capturing the following key information for each source:

- Author(s), year of publication, and study location
- Study aims and methodology
- Population characteristics, including carer and care recipient groups (if applicable)
- Intervention type, comparator (if any), and duration
- Outcome measures employed
- Key findings relevant to the review question(s)

This form will be iteratively refined as needed and will include both general and study‐specific fields if necessary to ensure consistency and comparability across the literature.

### 3.7 Data Analysis and Presentation

A narrative synthesis approach will be used to analyse the data across all categories that will take place for each study in tables and graphs.

## 4 Discussion

This scoping review will be the first, to our knowledge, to explore and examine the burden of DFUs specifically within the SA community residing within western countries. Given the disproportionately high prevalence of diabetes within this population, understanding the trajectory of diabetes‐related foot disease in this community, findings generated have the potential of benefiting the wider population affected by diabetes.

Existing studies suggest that the rates of DFUs and diabetes‐related amputation in SA are lower when compared to their White and Black counterparts [9]. This paradox is underexplored, and no current review comprehensively maps the existing evidence. We anticipate that by synthesising the available literature, this scoping review will provide clarity on how DFUs affect SA populations in a western context. Through identifying patterns and disparities in outcomes, as well as highlighting gaps in existing research.

This review employs a robust methodology grounded in the JBI framework and follows PRISMA‐ScR reporting guidelines. The use of standardised COS will enhance the comparability and clinical relevance of the included studies. Furthermore, registering the protocol with OSF ensures transparency, reproducibility, and allows for real‐time documentation of amendments.

As with all scoping reviews, this study will not assess the quality of the studies included, limiting its ability to comment on the strength of evidence [18]. There is also the potential for heterogeneity in how ethnicity is defined and reported across studies, which may limit comparability.

One anticipated limitation is the underreporting of diabetic foot complications among the South Asian population living in western countries. This may be due to cultural health‐seeking behaviours, delaying presentation to health care professionals, or language and socioeconomic barriers that prevent access to care. These factors would in turn result in lower visibility of complications in published literature. Therefore, we will note this as a contextual limitation when interpreting the findings of the review.

Despite these limitations, the breadth of the scoping review will offer a valuable overview of the existing evidence and identify critical areas for future research.

## Ethics Statement

The authors have nothing to report.

## Conflicts of Interest

The authors declare no conflicts of interest.

## Data Availability

The data that support the findings of this study are available from the corresponding author upon reasonable request.
